# Sex and Gender Differences on the Impact of Metabolism-Disrupting Chemicals on Obesity: A Systematic Review

**DOI:** 10.3390/nu16020181

**Published:** 2024-01-05

**Authors:** Massimo D’Archivio, Lucia Coppola, Roberta Masella, Alessia Tammaro, Cinzia La Rocca

**Affiliations:** Gender-Specific Prevention and Health Unit, Centre for Gender-Specific Medicine, Istituto Superiore di Sanità, 00161 Rome, Italy; massimo.darchivio@iss.it (M.D.); alessia.tammaro@guest.iss.it (A.T.); cinzia.larocca@iss.it (C.L.R.)

**Keywords:** sex, gender, metabolism-disrupting chemicals, obesity, endocrine disruptors, human exposure

## Abstract

Obesity represents an important public health concern, being one of the leading causes of death worldwide. It is a multifactorial disease with many underlying intertwined causes, including genetic, environmental and behavioral factors. Notably, metabolism-disrupting chemicals (MDCs) can alter the set point control of metabolism, affecting the development and function of the adipose tissue. Epidemiological studies have reported associations between human exposure to MDCs and several altered metabolic endpoints. It is also noteworthy that sex and gender represent important risk factors in the development of obesity. Different sex-related biological and physiological characteristics influence individual susceptibility, whereas gender represents a critical component in determining the different exposure scenarios. Although some advancements in the treatment of obesity have been achieved in preclinical and clinical studies, the obesity pandemic continues to increase worldwide. The present study performed a systematic review of recent studies considering the effects of MDCs on obesity, with a specific focus on sex- and gender-related responses. This review highlighted that MDCs could differently affect men and women at different stages of life even though the number of studies evaluating the association between obesity and MDC exposure in relation to sex and gender is still limited. This evidence should urge researchers to carry out studies considering sex and gender differences. This is essential for developing sex-/gender-tailored prevention strategies to improve public health policies and reduce exposure.

## 1. Introduction

Increasing overweight and obesity worldwide have created a significant burden for public health. Genetic factors and unhealthy lifestyles, namely excessive caloric consumption and sedentary life, are involved in the development of obesity and related diseases, such as type 2 diabetes mellitus (T2D), non-alcoholic fatty liver disease (NAFLD), cardiovascular disease, and some types of cancer [1]. Notably, recent evidence has suggested that some environmental chemicals, recognized as metabolism-disrupting chemicals (MDCs), can alter the set point control of metabolism, affecting the development and function of the adipose tissue (AT) and other organs like the liver, pancreas, and brain [2]. Consequently, the MDC hypothesis is gaining increasing attention as a factor contributing to obesity development [3].

The impact of chemical exposure on human health is highly influenced by both sex and gender. Different sex-related biological and physiological characteristics in men and women influence individual susceptibility, whereas gender represents a critical component in determining the different exposure scenarios. Indeed, gender relates to several socially constructed characteristics of women and men—such as norms, roles and relationships between groups of women and men [4].

The importance of integrating sex and gender analysis in research studies is well established; the most important funding agencies, such as the European Commission and the National Institutes of Health, have signed policy changes concerning this issue. A better understanding of the effects of MDCs on obesity in humans considering sex and gender analyses will lead to the development of sex-/gender-tailored prevention strategies, improving health and disease management. Within this frame, gender medicine highlights the interrelation between human health and the aspects of sex and gender, underlining variations in disease patterns between men and women in terms of prevention, clinical signs, therapeutic approach, prognosis, rehabilitation and psychological and social impact. In addition, the sex- and gender-tailored evaluation of MDC exposure may play a pivotal role in improving chemical risk assessment.

This review aims to provide a comprehensive summary of the most recent studies considering the effects of MDCs on obesity in humans, with a specific focus on sex- and gender-related responses. The final objective is to highlight the relevance of sex and gender as determinants of exposure in men and women, promoting the integration of this issue in epidemiological studies.

### 1.1. Obesity

Obesity represents an important public health concern, and it is one of the leading causes of death and disability worldwide and the fourth-highest independent cause of premature mortality [5]. It is a multifactorial disease with many underlying intertwined causes, including genetics, environmental and behavioral factors. While it is widely acknowledged that environmental factors, including a high-caloric food intake and sedentary lifestyle, contribute to obesity risk, it is also clear that genetics contribute substantially to determining an individual’s response to an ‘obesogenic environment’ [6], with some individuals genetically predisposed to gain weight more easily than others [7].

Obesity is characterized by abnormal or excessive fat accumulation, with adverse effects on human health. Besides the storage and distribution of energy functions, AT is an active endocrine organ producing numerous hormones and bioactive molecules known as adipokines, which play a key role in the maintenance of metabolic health, immune response and systemic inflammation. Healthy adipocytes, normally sensitive to insulin, produce anti-inflammatory adipokines [8]. The physiological production of adipokines requires the intact cellular machinery of mature adipocytes, particularly regarding mitochondrial respiration, and maintaining a balance between lipogenesis and lipolysis.

Conversely, during obesity, AT is characterized by increased visceral fat accumulation, leading to AT hypertrophy with the infiltration of pro-inflammatory M1 macrophages, as well as impaired adipogenesis, angiogenesis and lipolysis. The dysfunctional visceral AT secretes pro-inflammatory adipokines that influence the surrounding microenvironment, altering the regulation of glucose and lipid metabolism [9] and contributing to the establishment of chronic low-grade inflammation [10]. This condition may increase the prevalence of numerous obese-related disorders, such as T2D, NAFLD and cardiovascular diseases, including hypertension, atherosclerosis and coronary heart diseases [11,12,13,14]. Furthermore, the connection between obesity and cancer is increasingly evident, with breast, colon and uterine cancer being more prevalent in obese individuals [15]. This connection may be linked to the hormonal and inflammatory changes induced by obesity [16].

It is also noteworthy that sex and gender represent important risk factors in the development of obesity. Sex differences in body composition result in different rates of metabolic and cardiovascular alterations associated with obesity [17]. Indeed, women have a more favorable subcutaneous AT distribution, while men have predominantly accumulation of visceral AT [18]. These different patterns of body fat distribution may contribute to the different prevalence of obesity-related pathologies in men and women [19].

Several factors lead to gender disparities in obesity, including socioeconomic and sociocultural status [20]. Moreover, dietary habits and physical activity are strongly influenced by gender-related attitudes that promote different patterns of healthy or unhealthy lifestyles among women and men [21]. Women have a more pronounced trust in healthy diets and greater engagement in controlling body weight, and they select healthier food than men. On the other hand, men practice regular physical activity more frequently than women and modify their diet to reduce their body weight and improve their physical condition [22].

Although some advancements in the treatment of obesity have been achieved in preclinical and clinical studies, with dietary restriction and increased exercise remaining the most prescribed treatment, the obesity pandemic continues to increase worldwide due to its multifactorial etiology, including environmental factors [23].

### 1.2. Metabolism-Disrupting Chemicals

Despite environmental, nutritional and socioeconomic factors, increasing evidence has linked chemical exposure to the development of obesity and other metabolic and endocrine diseases [24].

Epidemiological studies have reported associations between human exposure to phthalates, organochlorine pesticides, polychlorinated biphenyls (PCBs) and bisphenol A (BPA) and several altered metabolic endpoints and outcomes, including increased body mass index (BMI) and serum lipid levels, as well as the onset of obesity, metabolic syndrome, insulin resistance and T2D [25,26,27,28,29]. In 2015, during a scientific international meeting on obesity and endocrine disruptors (EDs), defined as “exogenous substances that alter function(s) of the endocrine system and cause adverse health effects in an organism, or its progeny, or (sub) populations” [30], a consensus statement on the evidence that several EDs exhibit obesogenic effects and interfere with the metabolic processes in several organs and tissues, was issued. Therefore, the EDs displaying these characteristics were recognized as MDCs, including bisphenols, phthalates, pesticides, fungicides, PCBs, organochlorine, polybrominated and perfluorinated compounds. These chemicals are found in several consumer goods, such as cosmetics, plastic manufacture, food and beverage packaging and industrial chemicals [3,31,32,33]. The effects of MDC exposure depend on many factors, including biological, hormonal and physiological differences between women and men; and also social and cultural factors, as well as the timing of the exposure. Notably, several human developmental phases exhibit increased sensitivity, which has led to the definition of “windows of vulnerability and susceptibility”, which include pregnancy and early childhood, puberty and reproductive age [3,23,31,34]. Additionally, it has been observed that exposure to MDCs during development increases the susceptibility to obesity and its related diseases, although a second "hit", such as a high-fat or high-sugar diet, could be necessary for phenotypic expression to become evident [3].

The mechanisms through which MDCs exert their effects on human health are complex. One of the primary mechanisms involves endocrine disruption by the MDCs of estrogens, thyroid hormones and proliferator-activated receptors (PPARs), which play a key role in regulating metabolism and AT development. The disruption of the endocrine system can lead to metabolic dysregulation and alterations in fat cell differentiation and growth [31]. Moreover, MDCs can influence adipogenesis, increasing the formation and accumulation of adipocytes and, consequently, the storage of fat in the body [35,36]. Another critical mechanism involves appetite regulation. Some MDCs can affect the central nervous system and the appetite control centers of the brain, resulting in an altered sense of hunger and satiety. This disruption can contribute to overeating and weight gain [25,36,37]. In addition, effects on lipid metabolism, inflammation and oxidative stress have been observed [38,39].

Emerging research suggests that MDCs can induce epigenetic modifications that may alter the expression of genes regulating metabolism and fat storage, potentially transmitting obesity-related effects across generations [40,41]. It has been reported that MDCs represent a risk factor for the development of obesity-related diseases, such as cardiovascular diseases, including hypertension and heart [42]. Moreover, the disruption of the endocrine system by MDCs can also have implications for reproductive health and development, potentially influencing fertility and contributing to adverse pregnancy outcomes [43].

Therefore, since MDCs represent a concerning group of chemicals that can disrupt the metabolic balance of the body and contribute to obesity, an understanding of the mechanisms of action and the possible sex-related effects is essential for developing strategies for MDC identification and characterization in risk assessments.

## 2. Methodology

We conducted a systematic review following PRISMA guidelines and registered it in PROSPERO (ID registration: CRD42023493186). 

### 2.1. Search Strategy

We carried out a systematic search on PubMed and Web of Science to identify human studies that examine the associations between MDCs and obesity and related diseases, providing disaggregated data for sex. The publication date range considered was from May 2018 to June 2023 (five years). Our search strategy included the following key terms:

#1 (obesogen/s) or (obesity) or (obese) or (metabolic syndrome);

#2 (endocrine disruptor/s) or (phthalate/s) or (Polybrominated diphenyl ethers) or (PBDE) or (polycyclic aromatic hydrocarbon) or (PAH) or (Bisphenol) or (heavy metal) or (environmental);

#3 1 AND 2.

We entered the search terms individually and in combinations using the advanced search functionalities of both databases. Moreover, we searched the reference lists of the identified articles, but no further relevant articles were found.

### 2.2. Inclusion and Exclusion Criteria

This review was limited to human studies, such as cohort, cross-sectional and control studies, with MDC exposure data broken down by sex. Human studies without disaggregate data for sex or studies that focused only on female or male subjects were excluded. In vitro and animal studies, reviews, editorials, author’s reply, commentary or study protocol were excluded as well. Studies published in other languages apart from English and duplicate articles were also excluded.

### 2.3. Study Screening and Data Extraction

**Figure 1:**
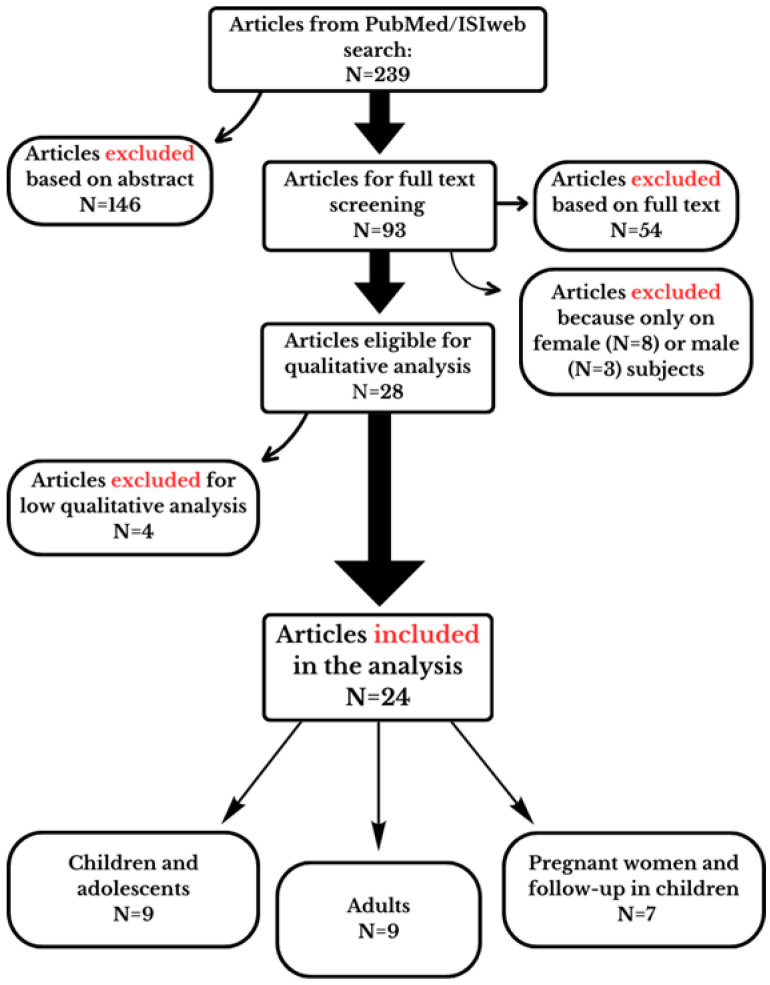
Flow chart illustrating the study selection process to identify eligible studies. The reasons for the exclusion in each step are also reported in the figure.

The initial screening of retrieved articles involved reviewing titles and abstracts based on eligible inclusion and exclusion criteria. Subsequently, full texts were examined for further validation, accuracy and analysis of data. Any disagreements were resolved through discussion among the authors to reach a consensus. The flow chart of the study screening is shown in Figure 1; the number of studies included or excluded in each step is also reported.

### 2.4. Study Quality Assessment

The risk of bias in each human study, including cross-sectional, cohort and case–control studies, was assessed according to the Office of Health Assessment and Translation (OHAT)’s risk-of-bias rating tool [44]. Possible sources of bias are evaluated via pre-defined questions in the OHAT tool that are addressed relative to human studies. The following questions were considered for rating the risk of bias: comparison group appropriateness (question N° 3), potential confounders (question N° 4), incomplete outcome data (question N° 7), confidence in exposure characterization (question N° 8), confidence in the outcome assessment (question N° 9) and the reporting of results (question N° 10). The answer to each question was assigned to one of the four following categories of risk of bias: definitely low (++), probably low (+), probably high (-; not reported, NR) or definitely high (--). Finally, a “3-tier system” was utilized to categorize studies into low, moderate and high risk of bias levels, mainly concerning the risk of bias relative to key elements related to questions 4, 8 and 9 according to the OHAT approach’s guidelines [45].

## 3. Results and Discussion

Two hundred and thirty-nine articles were initially identified and retrieved from databases and included in our assessment. After reviewing the abstract of each study, 146 studies were excluded, because 49 articles were on animal or in vitro studies; 46 were reviews, letters, commentaries or study protocols; 39 were irrelevant for exposure or/and outcome; 9 were duplicates; and 3 were not in the English language. The full-text articles of the remaining 93 studies were further assessed. Among these, 54 articles were excluded due to the lack of disaggregated data for sex or because they were irrelevant with respect to exposure, and 11 were excluded because only female or male subjects were analyzed. Twenty-eight studies were eligible for the qualitative analysis (Figure 1).

The studies were grouped into categories based on the population under investigation, i.e., children/adolescents, adults and birth cohort studies with pregnant women exposure and follow-ups with children. Notably, one study included data on both adults and children [46].

The characteristics of the studies selected are reported in Table 1, Table 2 and Table 3 for each group of population.

**Figure 2:**
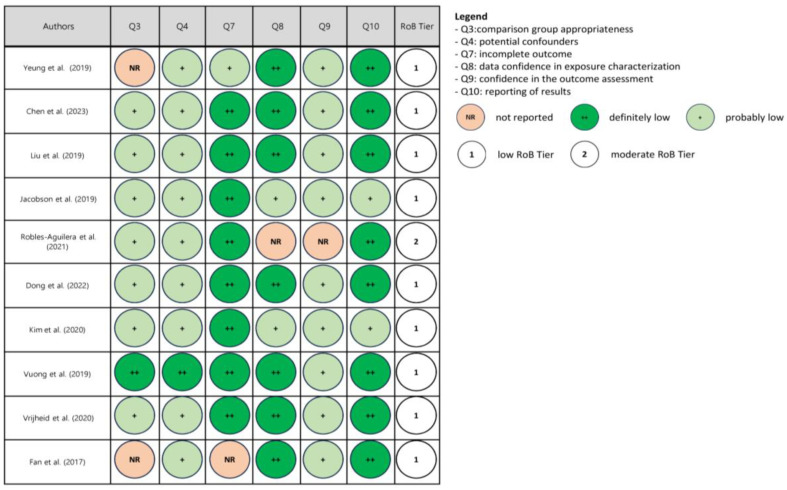
Risk of bias (RoB) assessment in children and adolescent studies [46,47,48,49,50,51,52,53,54,55].

**Figure 3:**
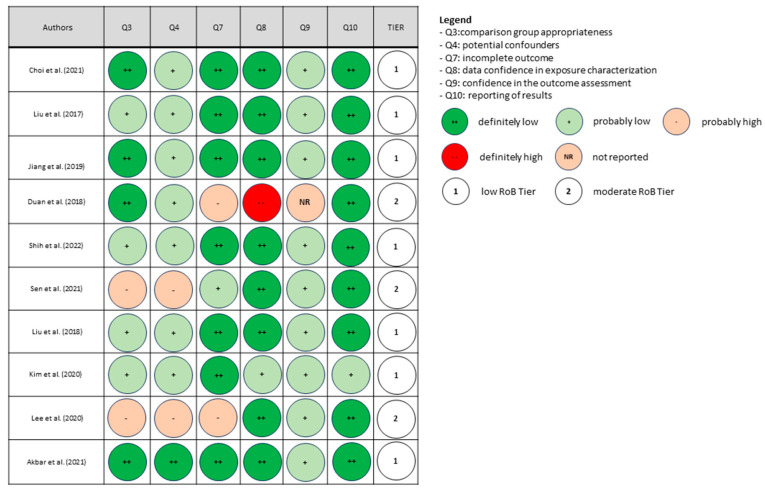
Risk of bias (RoB) assessment in studies of adults [46,56,57,58,59,60,61,62,63,64].

**Figure 4:**
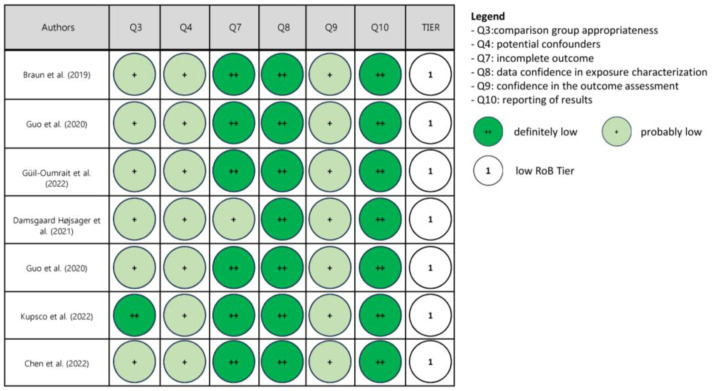
Risk of bias (RoB) assessment in studies in pregnant women and follow-up in children [65,66,67,68,69,70,71].

Upon risk of bias assessment, most studies were categorized in Tier 1 (low risk of bias), four in Tier 2 (moderate risk of bias) and none in Tier 3 (high risk of bias). The results of the assessment are reported in Figure 2, Figure 3 and Figure 4.

To ensure a rigorous analysis, the studies with a moderate risk of bias (Tier 2) were excluded. A total of 24 studies with a low risk of bias were included in the final discussion.

Given the heterogeneity in exposure to different chemicals and the several endpoints assessed, a narrative synthesis has been conducted [45].

### 3.1. Studies on Children and Adolescents

Nine studies provided disaggregated exposure data for male and female children and adolescents (Table 1). Seven studies had a cross-sectional study design: One was a case–control study, and one calculated exposure via dietary intake. Four studies analyzed plasticizers, such as bisphenol A, S and F; and phthalates (mono-methyl phthalate (MMP), mono-ethyl phthalate (MEP), mono-n-butyl phthalate (MBP), mono-iso-butyl phthalate (MiBP), mono-2-ethylhexyl phthalate (MEHP), mono-2-ethyl-5-oxohexyl phthalate (MEOHP) and mono-2-ethyl-5-hydroxyhexyl phthalate (MEHHP). One study evaluated conservative products, such as parabens (methyl, butyl, ethyl and propyl paraben). One study evaluated a class of persistent compounds, such as polybrominated diphenyl ether congener levels (28, 47, 99, 100 and 153 and their sum ΣPBDEs), and one focused on metals (lead, mercury and selenium). The measured endpoints, included several factors associated with obesity and metabolic syndrome, such as waist circumference, plasma cholesterol and triglycerides; fasting blood glucose; and blood pressure and insulin resistance. The sample size ranged from 206 [53] to 5404 [55], with approximately 50% of the subjects being boys in all the studies examined. The studies assessed exposure during childhood; most involved children and adolescents aged 6–17 years, and two studies involved infants from 0 to 3 years old [47,53]. Standardized protocols were used for anthropometric measurements, and the physiological parameters related to metabolic syndrome components were analyzed according to laboratory procedures. Appropriate detection methods were employed to quantify chemicals exposure levels: High-performance liquid chromatography–tandem mass spectrometry (HPLC-MS/MS) was used for bisphenols, phthalates and parabens in urine samples or perfluorooctane sulfonate (PFOS) and perfluorooctanoic acid (PFOA) in blood samples; inductively coupled plasma mass spectrometry (ICP-MS) was used for metals or gas chromatography/isotope dilution high-resolution mass spectrometry (GC-HRMS) was used for PBDEs in blood samples. Cross-sectional studies reported the association between bisphenols and phthalates, used in the manufacture of polycarbonate plastics and resins, and obesity in children. 

Data from the NHANES survey conducted by the National Center for Health Statistics of the Centers for Disease Control and Prevention from 2013 to 2014 and from 2015 to 2016 provided an estimation of the association between BPA, bisphenol S (BPS) and F (BPF) exposure and overweight, severe obesity and abdominal obesity in children and adolescents aged 6 to 19 years [49,50]. The determined endpoints included body mass index (BMI) z-scores, overweight and abdominal obesity defined as a waist circumference/height ratio. Both studies indicated that BPA exposure was associated with general and abdominal obesity in boys but not in girls. These studies also suggested an association between obesity and BPS [50] or BPF exposure [49]; interestingly, in both cases, the association was more prominent in boys than in girls. In addition, Liu et al. showed that BPA and BPF urinary levels were not influenced by the different dietary habits adopted by boys and girls. Interestingly, also for other bisphenols, such as BPAF, the association with obesity was greater in boys than in girls [48].

This sex-related association seems to be unaffected by the child’s age; indeed, stronger associations between BPA exposure and weight and BMI were also observed in younger children aged 4 months to 3 years [47]. As for bisphenols, positive associations with overweight and obesity were found for phthalates in boys and girls, with boys having a higher prevalence of overweight and obesity [52]. Liu et al. 2019 [49] evaluated aspects linked to gender, such as race/ethnicity, education level, the poverty/income ratio of the family (PIR) and behavioral factors, including time spent watching television and tobacco smoke exposure. Although the results showed that BPA and BPS concentrations were inversely associated with PIR, the analysis did not consider the stratification by sex [49].

Overall, all these studies showed boys to be more susceptible than girls to the obesogenic effects of bisphenols and phthalates. At present, since very few studies evaluated the possible influence of gender on the effects of MDCs, the cause of different responses between males and females can be ascribed to biological factors. For example, boys showed higher metabolic efficiency in transforming Di(2-ethylhexyl) phthalate (DEHP) into its metabolites, and this is probably due to the different specific P450 enzyme activities [72].

Concerning child exposure to persistent compounds, the data showed no clear pattern; only one study showed weaker associations for boys than girls for PFOS and PFOA [47], and this was not confirmed by other authors [54]. In contrast, differences between boys and girls were not observed in the association of those compounds with adiposity [53,54].

Only one cross-sectional study concerned the effects of metallic elements, including lead, mercury, selenium, manganese, copper and zinc, on obesity in children and adolescents (6–19 years). The results showed that metals were positively correlated with cholesterol levels in both boys and girls. The girls showed a positive correlation with mercury and selenium exposure, while the boys had a positive correlation with zinc exposure, clearly highlighting a sex-related response to the same obesogenic factor [55]. To date, the mechanisms underlying the association between selected metallic elements and childhood obesity, as well as any potential differences between males and females, remain to be investigated. Furthermore, it must also be emphasized that scientific data suggested a prevalence of obesity among boys compared to girls aged 5–19 years [73]. It has been hypothesized that these differences may be influenced by different fat body compositions and sexual hormones [74,75], as well as gender-related effects, such as sociocultural influences and parental feeding practices [76,77]. These aspects may contribute differently to the definition of the impact of MDC exposure in children.

The overview of contaminants associated with obesity outcomes in a sex-specific manner is reported in Table 4. 

### 3.2. Studies on Adults

Eight cross-sectional studies and one case–control study were focused on adults (18–80 years), categorizing participants into women and men (Table 2). Four studies investigated bisphenols; the other ones focused on phthalates, parabens, perfluorinated alkyl substances (PFAS), persistent organic pollutants and metals. A cluster of metabolic factors, including abdominal obesity, high blood pressure, impaired fasting glucose, high triglyceride levels, and low high-density lipoprotein (HDL) and cholesterol levels, was evaluated. In each study, chemicals were determined in an appropriate biological matrix (blood, plasma and urine) with specific analytical methods (HPLC-MS/MS; GC-HRMS; ICP-MS). The sample size ranged from 621 [62] to 4314 [56]; in all studies, men comprised about 50%.

Several studies showed a stronger association in men than in women between phthalate metabolites, i.e., MEP, bisphenols (BPA and BPS) or parabens, and risk factors for metabolic syndrome, i.e., general and abdominal obesity, dyslipoproteinemia, hypercholesterolemia and hypertension [46,57,58,60]. This sex-specific response to MDC exposure may be due to the higher expression of estrogen in women, which plays a protective role in several components of metabolic diseases and the cardiovascular system, including blood pressure and hypertension [78]. Moreover, estrogen has a key role in the regulation of hepatic lipid synthesis, specifically in cholesterol biosynthesis, which is more active in women than in men, thus influencing the different impacts of MDC exposure in male and female subjects [79,80].

Two studies highlighted the relevance of gender factors, such as race/ethnicity or geographical areas, in relation to several metabolic factors and bisphenol exposure [56]. The first study examined the role of race/ethnicity in US adults in relation to the associations between bisphenol levels and obesity using the American National Health and Nutrition Examination Survey (NHANES, 2013–2014). The results showed that the association between BPA exposure and obesity was stronger in men and whites compared to women and non-whites, while differences were found for BPF and BPS exposure [57].

The second study investigated the relevance of geographical areas by comparing data between US (NHANES 2013–2016) and Korean adults (Korean National Environmental Health Survey (2015–2017)) [56]. Comparable associations between BPA and BMI were observed in both sexes of the US and Korean population. However, differences were highlighted for other bisphenols: BPS exposure showed a weak positive association with BMI in US men and Korean women, while BPF levels were associated with BMI only in US women. When considering different metabolic factors, such as HDL-C plasma levels and serum triglycerides affected by lipid metabolism disorders, BPA levels were negatively associated with HDL-C levels in both US and Korean men and positively associated with triglyceride (TG) levels in US and Korean adults, and they were higher in US men. Significant associations between BPF and HDL-C levels were detected only in US women. In Korean women, the initial association between BPF and TG levels was not confirmed after adjustments for all covariates, including demographic and socio-economic factors; and healthy behaviors, strengthening the possible relevant role of gender factors. Overall, these results highlight the need to take into consideration the impact of both gender factors and sex differences in environmental health studies.

The overview of contaminants associated with obesity outcomes in a sex-specific manner is reported in Table 4. 

### 3.3. Studies on Pregnant Women and Follow-Up in Children

Among the selected papers, seven birth cohort studies were considered, with pregnant women checked for chemical exposure and newborn children observed at different ages to evaluate the associations between prenatal exposure and obesity factors in childhood. The number of mother–child pairs, considering boys and girls, ranged from 260 [70] to 1015 [67], with enrolment performed in European, American and Chinese countries (Table 3). Three studies investigated plasticizers, including bisphenols and phthalates; one measured paraben; three examined PBDE.

Braun et al. examined the association between maternal urinary BPA concentrations during pregnancy and central adiposity in children aged from 1.9 to 6.2 years old. This comprehensive analysis included parameters such as waist circumference, subscapular skinfold thickness and BMI. It is crucial to note that the association concerning waist circumference and subscapular skinfold thickness was modulated by the child’s sex: In girls, maternal BPA exposure during pregnancy was linked to increased values of all parameters, whereas these associations were generally inconclusive or showed a slight inverse trend in boys [65]. Consistent with these findings, Guo et al. reported a positive association between prenatal BPA exposure and waist circumference only among girls, with no significant associations observed in boys [66]. Similarly, exposure to sunscreen agent benzophenone-3 (BP3) was associated with a higher BMI and diastolic blood pressure in girls [67]. With respect to prenatal exposure to parabens, boys were shown to be more susceptible than girls. In particular, exposure to n- butylparaben (BuP) increased the male total fat and android fat percentages [68].

PBDE congeners showed different associations with adiposity depending on the congeners and parameters considered. Prenatal exposure to BDE-154 was correlated with reductions in BMI z-scores and waist circumference in boys but not in girls. Boys with higher concentrations of BDE-153, BDE-100 and BDE-28 had higher adiposity measures and an increased risk of rapid growth than girls. Differently, girls showed a positive association between BDE-100 and BDE-153 and adiposity, while BDE-28 and BDE-47 showed a negative tendency [69].

Overall, the combined data suggest that prenatal exposure to PBDE, phenols, phthalates and parabens recognized as MDCs may affect sex-specific body fat distributions in children due to the chemical interaction with steroid glucocorticoid and PPAR receptors, which are involved in AT distribution and function [81,82]. Furthermore, the higher body fat percentage in girls at birth and during adolescence supports their increased vulnerability to MDC effects [83,84]. Hence, it is essential to consider the complex interplay between environmental exposure, child sex, maternal exposure and other factors when studying the effects of MDCs on childhood health outcomes.

The overview of contaminants associated with obesity outcomes in a sex-specific manner is reported in Table 4. 

## 4. Conclusions

This review highlighted that the number of studies evaluating the association between obesity and MDC exposure related to sex and gender is limited. However, the analysis of available studies clearly demonstrated that chemicals might differently affect men and women at different stages of life.

Nevertheless, the topic remains controversial since it cannot be said with certainty whether and how exposure to MDCs affects men or women more specifically and, thus, which sex could be more susceptible. Indeed, this review showed that the response in the two sexes could differ depending on the chemical analyzed as well. Moreover, the evaluation of the impact of exposure is more complex since it is known that people are not exposed to a single substance but to a mixture of chemicals. Therefore, what we observe may result from the combined effects of multiple chemicals: This represents an additional challenge for risk evaluation and tailored prevention strategies.

This evidence should urge researchers to carry out a greater number of studies that consider sex and gender components. The following are crucial for an appropriate evaluation of MDC impact: (i) include subjects of both sexes; (ii) analyze data in a disaggregated manner to identify potential sex-specific effects; and (iii) consider gender factors that can differently influence men and women. Moreover, understanding the mechanisms of action of MDCs and their effects on human health, considering sex and gender differences, is essential for developing tailored prevention strategies with the aim of reducing exposure and improving public health policies.

## Figures and Tables

**Table 1:** Selected studies on children and adolescents.

Authors	Country	Age Range	N of Subjects	Chemicals	Biological Samples	Endpoints
Yeung et al. [47]	USA	0–3	881 F; 969 M	Bisphenols	Blood	BMI, WFL
Chen et al. [48]	China	7	200 F; 226 M	Bisphenols	Urine	BMI, waist circumference, body fat percentage
Liu et al. [49]	USA	6–17	374 F; 371 M	Bisphenols	Urine	BMI, abdominal obesity
Jacobson et al. [50]	USA	6–19	894 F; 937 M	Bisphenols	Urine	BMI, abdominal obesity
Robles-Aguilera et al. [51]	Spain	12–16	273 F; 312 M	Bisphenols	Blood	BMI
Dong et al. [52]	China	7–13	168 F; 519 M	Phthalates	Urine	BMI
Kim et al. [46]	Canada	3–17	695 F; 723 M	Parabens	Urine	BMI, waist circumference, HDL cholesterol, triglycerides, fasting blood glucose and blood pressure
Vuong et al. [53]	USA	1, 2, 3, 5 and 8	114 F; 92 M	Polybrominated diphenyl ethers	Blood	BMI, waist circumference and body fat percentage
Vrijheid et al. [54]	6 European Countries	6–11	711 M; 590 F	Organochlorine compounds, polybrominated diphenyl ethers, per- and polyfluoroalkyl substances, metals and elements, phthalate metabolites, phenols, organophosphates, pesticide metabolites, cotinine	Urine	BMI, waist circumference, skinfold thickness and body fat mass
Fan et al. [55]	USA	6–19	2659 F; 2745 M	Heavy metals	Blood	BMI, triglyceride, cholesterol, low-density lipoprotein and HOMA-IR

Abbreviations: F, females; M, males; BMI: body mass index; WFL: weight for length; HDL: high-density lipoprotein; HOMA-IR: Homeostasis Model Assessment of Insulin Resistance.

**Table 2:** Selected studies on adults.

Authors	Country	Age Range	N of Subjects	Chemicals	Biological Samples	Endpoints
Choi et al. [56]	USA; Republic of Korea	20–70	USA: 550 F; 496 M Republic of Korea: 1764 F; 1504 M	Bisphenols	Urine	BMI, HDL cholesterol and triglyceride levels
Liu et al. [57]	USA	≥20	789 F; 732 M	Bisphenols	Urine	BMI and abdominal obesity
Jiang et al. [58]	China	56 ± 9	582 F; 855 M	Bisphenols	Urine	Blood pressure
Duan et al. [59]	China	51–58	Controls: 156 F; 95 M Cases: 106 F; 145 M	Bisphenols	Urine	Type 2 Diabetes Mellitus
Shih et al. [60]	Taiwan	30–70	693 F; 644 M	Phthalates	Urine	Waist circumference, blood pressure, fasting blood glucose, fasting serum triglycerides, high-density lipid cholesterol
Sen et al. [61]	USA	18–75	70 F; 35 M	Perfluorooctanesulfonate compounds, perfluorohexanesulfonic acid, polyfluorinated compounds	Plasma	Liver fat content, HOMA-IR
Liu et al. [62]	USA	30–70	384 F; 237 M	Perfluorooctanesulfonic acid, polyfluorinated compounds, perfluorohexanesulfonic acid, perfluorodecanoic acid	Plasma	Body weight and RMR
Kim et al. [46]	Canada	>18	568 F; 568 M	Parabens	Urine	BMI, waist circumference, HDL cholesterol, triglycerides, fasting blood glucose and blood pressure
Lee et al. [63]	Republic of Korea	19–80	3687 F; 2767 M	Mercury	Blood and Urine	Total cholesterol, HDL, and triglycerides, hepatic enzymes (ALT, AST, GGT)
Akbar et al. [64]	Canada	20–65	415 F; 280 M	10 persistent organic pollutants and toxic metals	Plasma	BMI, waist circumference and body fat percentage

Abbreviations: F, females; M: males; BMI: body mass index; HDL: high-density lipoprotein; HOMA-IR: Homeostasis Model Assessment of Insulin Resistance; RMR: resting metabolic rate; ALT: alanine transaminase; AST: aspartate aminotransferase; GGT: gamma-glutamyl transferase.

**Table 3:** Selected studies concerning pregnant women and follow-up in children.

Authors	Country	Age Range	N of Subjects	Chemicals	Biological Samples	Endpoints in Children
Braun et al. [65]	Canada	Mothers: 18–35 Children: 1.9–6.2	Mothers: 719 Children: 363 F; 356 M	Bisphenols	Urine	BMI, waist circumference and subscapular skinfold thickness
Guo et al. [66]	China	Mothers: 17 to ≥35 Children: 3 and 7	Mothers: 363 Children: 157 F; 206 M	Bisphenols	Urine	BMI, waist circumference and skinfold thickness
Güil-Oumrait et al. [67]	Spain	Mothers: 31 Children: 11	Mothers: 1015 Children: 500 F; 515 M	Phthalates and phenols	Urine	BMI, systolic and diastolic blood pressure
Højsager et al. [68]	Denmark	Mothers: 15–49 Children: 7	Mothers: 312 Children: 156 F; 156 M	Parabens	Urine	BMI, fat percentage, android and gynoid fat percentage
Guo et al. [69]	China	Mothers:17–35 Children: 7	Mothers: 318 Children: 138 F; 180 M	Polybrominated diphenyl ethers	Umbilical cord serum	BMI, waist circumference and skinfold thickness
Kupsco et al. [70]	USA	Mothers: 25 Children: 5–11	Mothers: 260 Children: 145 F; 115 M	Polybrominated diphenyl ethers	Umbilical cord blood and peripheral blood	BMI
Chen et al. [71]	China	Mothers: <25–≥35 Children: 0–6	Mothers: 340 Children: 148 F; 192 M	Polybrominated diphenyl ethers	Umbilical cord blood	BMI, arm circumference and waist circumference

Abbreviations: F, females; M, males; BMI: body mass index.

**Table 4:** Overview of the contaminants associated with obesity outcomes in a sex-related manner.

Subjects	Contaminants	Obesity Outcomes	Sex	References
Children	BPA, BPS, BPF	Abdominal obesity and overweight	M	Liu et al. [49] Jacobson et al. [50]
Children	BPAF	Obesity and overweight	M	Chen et al. [48]
Children	Phtalathes	obesity	M	Dong et al. [52]
Children	PFOS, PFOA	Adiposity parameters	M	Yeung et al. [47]
Children	Mercury/selenium	Cholesterol level	F	Fan et al. [55]
Children	Zinc	Cholesterol level	M	Fan et al. [55]
Adults	MEP, BPA, BPS, parabens	Obesity	M	Kim et al. [46], Liu et al. [57], Jiang et al. [58], Shih et al. [60]
Adults	BPF	BMI and HDL-C levels	F	Choi et al. [56]
Adults	BPA	HDL-C levels	M	Choi et al. [56]
Pregnant women and follow-up in children	BPA	Adiposity parameters, obesity	F	Braun et al. [65], Guo et al. [66]
Pregnant women and follow-up in children	BP3	BMI	F	Guil-Oumrait et al. [67]
Pregnant women and follow-up in children	Parabens	Adiposity parameters	M	Højsager et al. [68]
Pregnant women and follow-up in children	BDE-154, BDE-153, BDE-100 BDE-28	Adiposity parameters	M	Guo et al. [69]
Pregnant women and follow-up in children	BDE-153, BDE-100	Adiposity parameters	F	Guo et al. [69]

Abbreviations: M: males; F: females; BPA: bisphenol A; BPS: bisphenol S; BPF: bisphenol F; BPAF: bisphenol AF; PFOS: perfluorooctane sulfonate; PFOA: perfluorooctanoic acid; MEP: mono-ethyl phthalate; BP3: benzophenone-3; BMI: body mass index; BDE-28: 2,4,4′-Tribromodiphenyl ether; BDE-100: 2,2′,4,4′,6-Pentabromodiphenyl ether; BDE-153: 2,2′,4,4′,5,5′-Hexabromodiphenyl ether; BDE-154: 2,2′,4,4′,5,6′-Hexabromodiphenyl ether.
